# Genes related to osmoregulation and antioxidation play important roles in the response of *Trollius chinensis* seedlings to saline-alkali stress

**DOI:** 10.3389/fpls.2023.1080504

**Published:** 2023-01-26

**Authors:** Rongmiao Hou, Lizhi Yang, Tana Wuyun, Shiyao Chen, Lu Zhang

**Affiliations:** ^1^ College of Landscape and Architecture, Zhejiang A&F University, Hangzhou, China; ^2^ College of Horticulture and Landscape Architecture, Northeast Agricultural University, Harbin, China; ^3^ Institute of Agricultural and Environmental Sciences, Estonian University of Life Sciences, Tartu, Estonia; ^4^ Zhejiang Provincial Key Laboratory of Germplasm Innovation and Utilization for Garden Plants, Zhejiang A&F University, Hangzhou, China

**Keywords:** salinity and alkalinity, *Trollius chinensis*, osmoregulation, antioxidant defense, reactive oxygen species, transcriptome sequencing

## Abstract

Saline-alkali stress is one of the main abiotic stress factors affecting plant growth and development. *Trollius chinensis* is a perennial herbal medicinal plant with high values for garden application. However, its response and tolerance to saline-alkali stress is unclear. In this study, we mixed four salts (NaCl: Na_2_SO_4_: NaHCO_3_: Na_2_CO_3_) with a concentration ratio of 1:9:9:1, and applied low (40 and 80 mM) and high (120 and 160 mM) saline-alkali stress to analyze osmotic regulation substances, antioxidant systems and the gene expression of *T. chinensis*. Along with higher saline-alkali stress, the leaf relative water content (RWC) started to decrease only from high stress, while the malondialdehyde (MDA) content in leaves decreased continuously, and the contents of proline (Pro), soluble sugar (SS) and soluble protein (SP) increased compared with control. The activities of antioxidant enzymes and the contents of non-enzymatic antioxidants were increased positively with the accumulation of superoxide anion (O_2_^•–^) and hydrogen peroxide (H_2_O_2_). For instance, the ascorbic acid-glutathione (AsA-GSH) cycle was enhanced in *T. chinensis* seedling leaves subject to saline-alkali stress. Principal Component Analysis (PCA) indicates that MDA, Pro, SS, SP, H_2_O_2_, O_2_^•–^, and GSH are important indexes to evaluate the response and tolerance of *T. chinensis* to saline-alkali stress. Through RNA-Seq, a total of 474 differentially expressed genes (DEGs) were found in plant under low saline-alkaline stress (40 mM, MSA1) *vs*. control. Among them, 364 genes were up-regulated and 110 genes were down-regulated. DEGs were extensively enriched in carbohydrate transport, transferase activity, zeatin biosynthesis, ABC transporters, and spliceosome. The transcription factor family MYB, BZIP, WRKY, and NAC were related to its saline-alkali tolerance. In addition, some DEGs encode key enzymes in the processes of osmoregulation and antioxidation, including betaine aldehyde dehydrogenase (BADH), inositol monophosphatase (IMP), chloroperoxidase (CPO), thioredoxin (Trx), and germin-like protein (GLPs) were found. Overall, these findings provide new insights into the physiological changes and molecular mechanism of *T. chinensis* to saline-alkali stress and lay a foundation for application of *T. chinensis* in saline-alkali environment.

## 1 Introduction

Soil salinization and alkalization areas are about 930 million hectares, accounting for about 7% of the total land area in the world (Munns, 2002). Globally, salinity damages more than 800 million hectares of agricultural land. In China, about 25% of agricultural area is comprised of saline-alkaline soils, which are underutilized (Liu and Wang, 2021). It has been well documented that the main soil salinity type in China is sodium carbonate, in which Na^+^, HCO_3_^-^, CO_3_^2-^ are the main salt ionomers and the pH value is generally above 8.5 (Wang et al., 2009; Zhang et al., 2020). Plant growth and agricultural output are hampered by the sodic, high-pH, and salt-rich soils (An et al., 2016). Therefore, how to develop and utilize saline-alkali soil has become a research hotspot.

Osmotic imbalance, excessive Na^+^ accumulation, and oxidative stress often occur in plants under single salt stress (Wei et al., 2017). However, the salt composition in saline-alkali soil is very complex, and it can also induce elevated pH disturbances through HCO_3_^-^ and CO_3_^2-^, resulting in physiological changes (Song et al., 2017). Under saline-alkali stress, plant membrane permeability is disrupted due to cell water loss and the production of reactive oxygen species (ROS) (Miller et al., 2010), therefore, the physiological and biochemical activities of plant were inhibited. Some plants have developed a series of saline-alkali tolerance mechanisms, including osmoregulation and antioxidant defense (Huang et al., 2014). Osmoprotectants, such as proline (Pro) and soluble sugar (SS), are accumulated to maintain osmotic balance under stress. Moreover, antioxidant defense processes involving antioxidant enzymes (SOD and POD) and ascorbate-glutathione (AsA-GSH) cycle system are promoted to scavenge stress-induced reactive oxygen species (Raziq et al., 2022). At present, one potent method to decipher regulatory networks of genes is transcriptome analysis, which considerably contributed to the discovery of multiple genes that regulate saline-alkali plants. For instance, transcriptome analysis was conducted to reveal the regulatory mechanism of a salt-tolerant plant *Achnatherum splendens*, and the results showed an enrichment of differentially expressed genes (DEGs) in the “redox” and “transcription factor activity” of Gene Ontology (GO) categories, and the transcription factor families, including MYBs, AP2/ERF, bHLH, bZIPs, NAC, etc. took part in the stress regulation (Liu et al., 2016). It has also been reported that the response of Rice to salt stress involved several signaling components, transcription factors and functional genes that directly mediated osmotic regulation, antioxidant and ion homeostasis (Ponce et al., 2021).

*Trollius chinensis* is a plant with high ornamental value and multiple medicinal values. It is distributed in the temperate and cold temperate areas of the northern hemisphere, from Southern Siberia to Southern Russian Far East and Northern China. Several studies have investigated its stress resistance, including drought and heat stress. However, to date, its regulatory mechanisms for stress tolerance are poorly understood, and no report has emerged to reveal the mechanisms underlying the tolerance of *T. chinensis* to saline-alkali stress. To examine the osmotic regulation and antioxidant capacity of *T. chinensis* leaves, diverse saline-alkaline environments were reproduced by combining various salt kinds and amounts in the present study. Together with physiological indicators, we investigate the molecular regulatory mechanisms of *T. chinensis* under saline-alkali stress by RNA-Seq. Our study provides a theoretical basis for molecular breeding of *T. chinensis* with strong resistance to saline-alkali and has important implication for cultivation of *T. chinensis* in saline-alkali land.

## 2 Materials and methods

### 2.1 Plant materials

*T. chinensis* seedlings were purchased from Jinlianchuan Herbal Medicine Planting company, Inner Mongolia, China, and planted in 10 cm diameter plastic pots containing peat soil and vermiculite (3:1, v:v). Seedlings were placed in the greenhouse of Northeast Agricultural University. The greenhouse conditions were controlled at a temperature of 23-25°C/16-18°C (day/night), a photoperiod of 12h with an average diurnal light intensity of ca. 600 µmol·m^-2^·s^-1^, and a humidity of 65%-75%. The seedlings were watered every two days to avoid water deficit.

When they had 5-8 leaves, seedlings with similar sizes were selected for treatments. The roots were cleaned softly using tap water to remove soil and then were transplanted into plastic pots (diameter of 12 cm) filled with sand and vermiculite (2:1, v:v). Then, the seedlings were watered once a week with Hoagland’s full nutrient solution for two weeks, and the rest of the time was watered with distilled water. After that, stress treatments were conducted as follows.

### 2.2 Saline-alkali treatment

Saline-alkaline stress (MSA) was simulated by mixing four salts (NaCl: Na_2_SO_4_: NaHCO_3_: Na_2_CO_3_) with a concentration ratio of 1:9:9:1. According to preliminary experimental results, we screened four saline-alkali concentration (40 mM, 80 mM, 120 mM, and 160 mM) to carry out the experiment. In total, there are five treatment groups, in which the salt compositions of the treatments were listed in **Table 1**. Every three days, from 17:00 to 18:00, the control group was watered with Hoagland solution only, while the treatment group was watered with Hoagland solution with the corresponding salt. To standardize daily light exposure, nine pots of seedlings from each treatment were randomly relocated, and evaporated water was replenished daily after weighing. To prevent a salt shock response, the saline-alkaline solution was administered at a rate of 40 mM each day until the maximum concentration was attained, at which time day 0 was established. Experiment was ended when the visible injury of the MSA4 group reached 80% of total leaf area (day 12), then leaf samples were taken in the next morning (day 13). Part of samples was used for determining physiological parameters, and the remaining part was frozen in liquid nitrogen and stored in -80°C refrigerator for RNA-Seq and qRT-PCR validation.

**Table 1:** Salt composition and pH of each treatment.

Treatent	NaCl (mM)	Na_2_SO_4_ (mM)	NaHCO_3_ (mM)	Na_2_CO_3_ (mM)	Total concentration (mM)	pH
Control	0	0	0	0	0	6.51
MSA1	2	18	18	2	40	8.38
MSA2	4	36	36	4	80	8.65
MSA3	6	54	54	6	120	8.87
MSA4	8	72	72	8	160	8.93

### 2.3 Measurement of physiological parameters

#### 2.3.1 Measurement of the relative water content

The relative water content (RWC) of leaves was determined as follows: weighing the fresh weight (Wf) and then immersing the sample in distilled water for eight hours. After drying on filter paper and weighing (Wt), leaves were dried at 65°C until the weight stable for determination of dry weight (Wd). The RWC is calculated as (Wf-Wd)/(Wt-Wd)×100% (Barrs and Weatherley, 1962).

#### 2.3.2 Measurement of membrane lipid peroxidation

Leaf malondialdehyde (MDA) content was measured using thiobarbituric acid (TBA) chromogenic method (Wang et al., 2021): 0.15 gof leaves were homogenized with 10% TCA and centrifuged at 1,341 *g* for 10 min. After cooling, 1 mL of 0.6% TBA-containing reaction mixture were mixed with 1 mL of the supernatant then underwent 15-min 100°C heating and 20-min 1,341 *g* centrifugation. At three distinct phases (450, 532, and 600 nm), we assessed supernatant absorbance.

#### 2.3.3 Measurement of osmotic substances

Free proline (Pro) content was determined using ninhydrin method (Nakhaie et al., 2020) with the absorption value at 520 nm: 0.2 g of frozen leaf samples were homogenized with 3% sulfosalicylic acid (5 mL); after 10 min, they were extracted from a boiling water bath, cooled, and eventually underwent 1,006 *g* centrifugation for 10 min. 2 mL acetic acid, 3 mL acidic ninhydrin, and 2 mL supernatant were mixed, and heated for 40 min at 100°C, then cooled and extracted with 5 mL toluene.

Soluble sugar (SS) content was determined using anthrone method (Buysse and Merckx, 1993) with the absorption value at 620 nm: 0.15 g of frozen leaves were homogenized at 80°C in a water bath for 30 min with 7 mL of 80% ethanol. After 10 min of 1,006 *g* centrifugation, the process of extracting the supernatant was repeated for 3-4 times. The supernatant of 1 mL was mixed with 5 mL anthrone-sulfuric for analysis.

Soluble protein (SP) content was measured using Coomassie brilliant blue G-250 staining (Wang et al., 2021) at 595 nm: 0.15 g of frozen leaf samples were added to 1.5 mL of phosphate buffer (50 mM, pH 7.8), ground in an ice bath and centrifuged (13,900, 20 min). 1 mL of the supernatant was mixed thoroughly with 5 mL G-250, and after 2 min, the absorption of solution was determined.

#### 2.3.4 Measurement of ROS accumulation

Hydrogen peroxide content was measured by titanium sulfate method (Li et al., 2012) with some modifications: The frozen leaf was extracted with acetone and underwent 1,677*g* centrifugation for 300 s prior to mixing with 5% titanium sulfate and 0.2 mL of ammonia water concentrate. After the precipitate was formed, the solution was centrifuged at 1,677 *g* for 5 min to discard the supernatant, then the precipitate was washed with acetone for three times, and 2 M concentrated sulfuric acid was added. After dissolving, the absorption value was determined at 415 nm.

Superoxide anion (O_2_^•–^) content was measured by hydroxylamine hydrochloride method (Elstner and Heupel, 1976) with minor modifications: Mix the frozen leaves’ supernatant with phosphate buffer (50 mM, pH 7.8) and 10 mM hydroxylamine hydrochloride for about 1 h at room temperature. P-aminobenzenesulfonic acid (17 mM) and α-naphthylamine (7 mM) were added, and water bathed at 30°C for 30 min. Finally, the absorbance was checked at 530 nm.

#### 2.3.5 Measurement of activities of antioxidant enzymes

We assessed activity of superoxide dismutase (SOD) using NBT photochemical reduction that is inhibited by 560 nm (Giannopolitis and Ries, 1977). The unit (U) was defined as the amount of enzyme that inhibits the photoreduction of NBT by 50% and SOD activity is expressed as U g^-1^ (FM).

Peroxidase (POD) activity was measured by determining the oxidation rate of guaiacol substrate induced by H_2_O_2_ at 470 nm per min (Zhang et al., 2015). The unit (U) was the enzyme quantity oxidizing 1 µmol (guaiacol) per min per gram of sample (FM).

#### 2.3.6 Measurement of AsA-GSH cycle system

Ascorbic acid (AsA) was determined at 525 nm using the method described by Kampfenkel et al. (1995) with modifications: Frozen leaf was added with 5% TCA and centrifuged at 15,000 *g* for 10 min, and the supernatant was extracted in the dark. 0.2mL supernatant, NaH_2_PO_4_ (0.15M) and 0.2mL H_2_O were added in sequence to the sample tube, and mixed for 30s, then 10% TCA, 44% H_3_PO_4_, 4% 2,2’-Dipyridyl and 3% FeCl_3_ were added, and kept in a water bath at 37°C for 1h.

Glutathione (GSH) was measured by DTNB method (Kamencic et al., 2000) and the absorbance was measured at 412 nm. Frozen leaf samples (0.1 g) were homogenized with 5% TCA and centrifuged at 20,850 *g* for 20 min at 4°C. AS sample containers included supernatant (1 mL), phosphate buffer (1 mL, 0.1M, pH 7.7), and DTNB (0.5 mL, 4 mM). AC sample containers included a supernatant (1 mL) and phosphate buffers (1 mL, 0.1M, pH 6.8; 1 mL, 0.1 M, pH 7.7). The absorbance was measured after 10 min at a temperature of 25°C. Utilizing the standard curve, the reduced glutathione concentration was determined.

Using the method of Nakano and Asada (1981) and by observing the ascorbic acid oxidation rate at 290 nm, the activity of ascorbate peroxidase (APX) was measured. The fraction of enzyme extract solution included a mixture of phosphate buffer (50 mM, pH 7.0), 0.1 mM EDTA, 0.1 M H_2_O_2_ and 0.5 M ascorbate. One unit (U) was defined as the amount of enzyme oxidizing 1 μmol (ascorbate) per min per gram of sample (FM).

Glutathione reductase (GR) was determined using the method described by Foyer and Halliwell (1976) with modifications: The supernatant was added with phosphate buffer (0.1M pH 7.5, containing 0.1 mM EDTA) and GSSG (5 mM), then NADPH (4 mM) was added to start the reaction. GR activity was determined at 340 nm every 30 s for a total of two min. One unit (U) is the quantity of enzyme necessary to oxidize 1 µmol (NADPH) per min per gram of sample (FM).

### 2.4 Transcriptome assembly and analysis

To further explore the mechanism of saline-alkali tolerance of *T. chinensis*, leaves from three independent plants in both the control group and the treatment group with slight salt damage (MSA1) were selected for RNA-Seq. 1.5 μg RNA per leaf sample was used for the library preparation. Six libraries were constructed using NEBNext^®^ Ultra™ RNA Library Prep Kit for Illumina^®^ (NEB, USA) following manufacturer’s recommendations. The libraries were sequenced on an Illumina Novaseq 6000 platform by the Beijing Allwegene Technology Company Limited (Beijing, China) and paired-end 150 bp reads were generated. Trinity was used to construct clean reads after adaptor sequences and low-quality raw read sequences were eliminated (Grabherr et al., 2011). Clean data of the libraries were submitted to the Sequence Read Archive (SRA) in National Center for Biotechnology Information (NCBI) with the accession codes of PRJNA893832. CD-hit (Fu et al., 2012) was used to classify transcripts, corset was used to remove redundancy, and BUSCO software was used to evaluate the quality of splicing. Then, these sequences were annotated by commonly used databases, including NCBI protein non-redundant database (NR), NCBI nucleic acid sequence database (NT), Kyoto Encyclopedia of Genes and Genomes (KEGG) Ortholog (KO), Swiss-Prot, Pfam (database) protein family, eukaryotes orthologous genes database (KOG), and GO. The genes with *p*adj < 0.05 and |log_2_ (Fold Change)| > 1 were screened as differentially expressed genes (DEGs) between MSA1 and control. GO function and KEGG metabolic pathway enrichment analysis was performed on the DEGs.

### 2.5 Validation of gene expression using quantitative real-time PCR

Total RNA was extracted from the leaves of the control group and MSA1 group by the RNA kit DP432 (Tiangen Biochemical Company, Beijing), and cDNA was synthesized by reverse transcription using the HiScript III 1^st^ Strand cDNA Synthesis Kit (Vazyme, Nanjing), and Primer Premier 6.0 (Premier Biosoft Inc, Canada) was used to design primers, and synthesized by Shanghai Sanong (**Table S1**) *Actin* was used as an internal reference gene. AceQ Universal SYBR qPCR Master Mix (Vazyme, Nanjing) was used for qRT-PCR on the ABI 7500 (Applied Biosystems) using the following reaction program: 95°C for 10 min; 40 PCR cycles (95°C for 15 s and 60°C for 60 s). Using the 2^-ΔΔCt^ relative quantification approach, the expression ratio was calculated (Livak and Schmittgen, 2001). Three independent biological replicates were analyzed for each sample.

### 2.6 Data analysis

The data were analyzed with SPSS 26.0 (SPSS, Chicago, USA) to conduct one-way ANOVA for physiological characteristics. All data are presented as means ± standard errors (SEs) of three replicates, and the significant differences were determined by the Duncan’s multiple range test. Pearson’s correlation among physiological parameters were analyzed. Histograms were drawn with Graphpad 7.0 (Graphpad dotmatics, San Diego, USA), and Origin 2021 (OriginLab, Northampton, USA) was used to conduct correlation analysis and principal component analysis (PCA). The relative change of physiological parameters between treatment and control was calculated as (Mean_treatment_- Mean_control_)/Mean_control_ × 100%.

## 3 Results

### 3.1 Leaf RWC and MDA content

**Figure 1:**
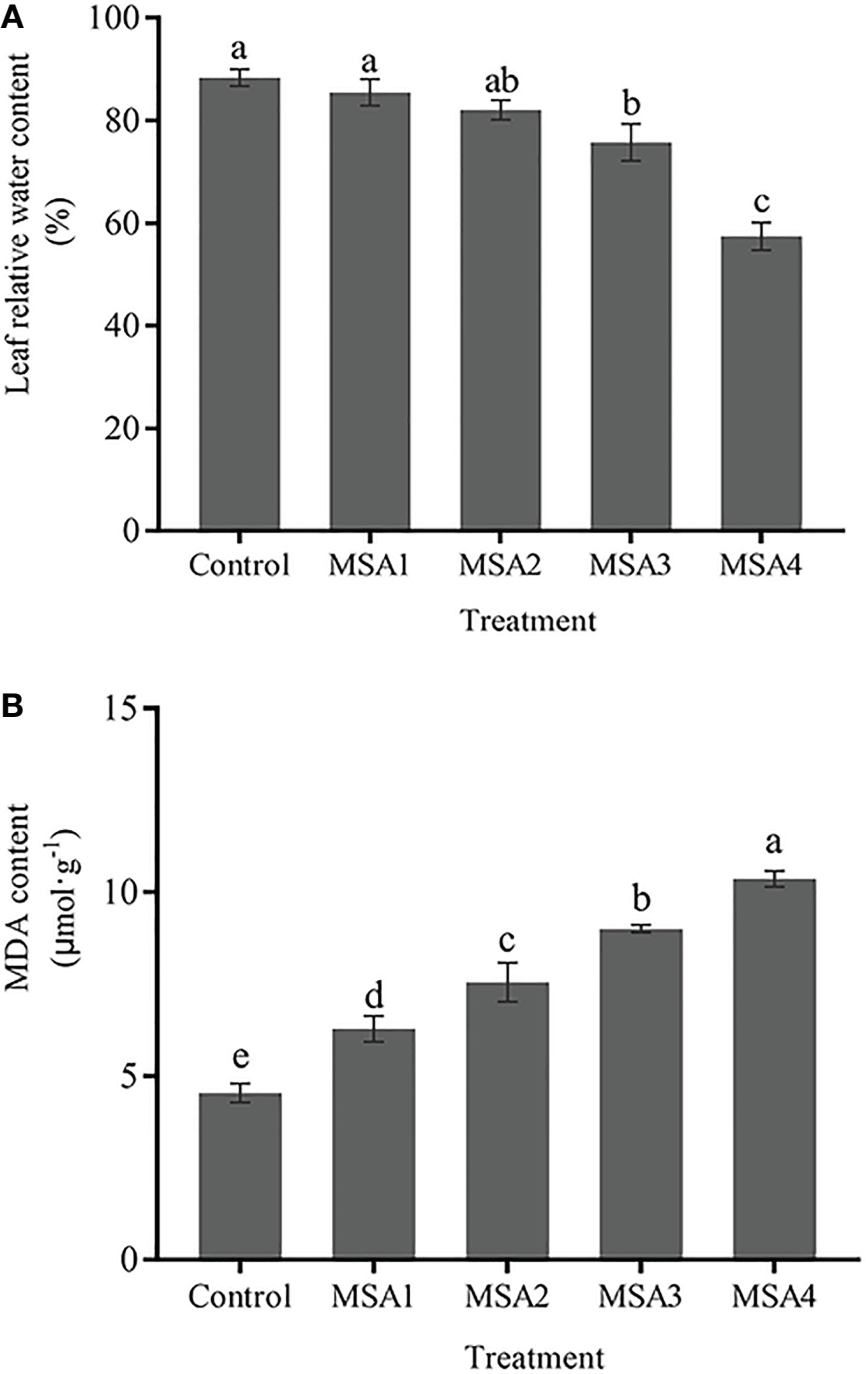
Changes in the leaf relative water content **(A)** and MDA content **(B)** of *Trollius chinensis* under saline-alkaline stress. Control, 0 Mm; MSA1, 40 mM; MSA2, 80 mM; MSA3, 120 mM; MSA4, 160 mM. Values are the means ± standard errors (SEs). Means followed by different letters indicate significantly difference between treatments at *p <* 0.05 according to Duncan’s test. The same as below.

No significant difference was found in leaf RWC between the low saline-alkali (MSA1 and MSA2) treatment groups and control (*p > 0.05*), while the difference between the high saline-alkali (MSA3 and MSA4) treatment groups and control was significant (*p <* 0.05) (**Figure 1A**). The leaf RWC reached the lowest value in the MSA4, which was significantly lower than that in the control (-34.99%, *p <* 0.05). However, the content of MDA in leaves increased continuously (**Figure 1B**), and the difference between each treatment group and control was significant (*p <* 0.05). The highest value was reached in MSA4, which was 2.28-fold higher than that in control (*p <* 0.01).

### 3.2 Osmotic regulators content

**Figure 2:**
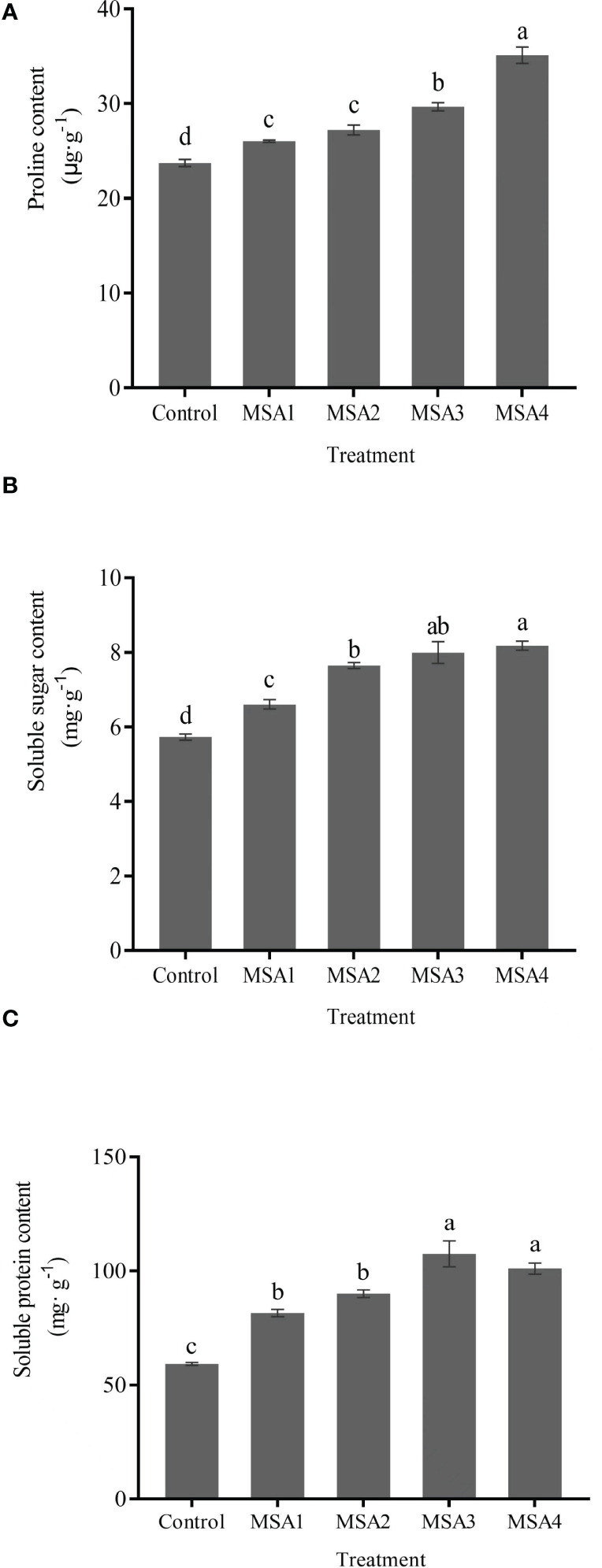
Changes in the osmoregulation substances contents of Trollius chinensis leaves upon saline-alkaline stress. **(A)**, Proline content; **(B)**, Soluble sugar content; **(C)**, Soluble protein content.

Differences of osmotic regulators content in leaves between each MSA treatment and control were significant (*p <* 0.05). The content of Pro and SS increased most in MSA4 treatment with 47.99% and 42.79%, respectively compared with control. Different from the changes in Pro and SS content, SP content reached the highest value in MSA3 treatment (**Figure 2**), which was increased by 81.33% compared with control, while slightly decreased in MSA4 treatment, but not significantly different from MSA3 treatment (*p > 0.05*).

### 3.3 ROS accumulation

**Figure 3:**
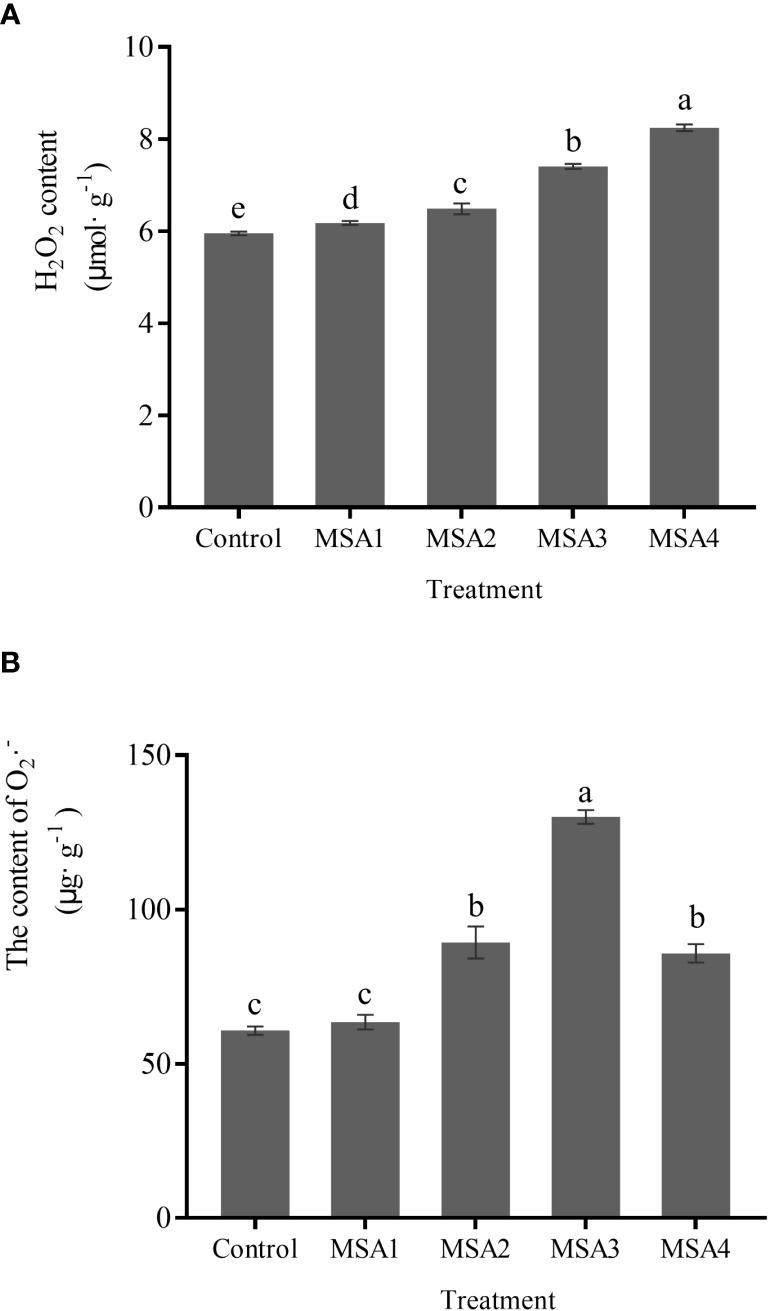
Changes in the contents of reactive oxygen species in *Trollius chinensis* leaves upon saline-alkaline stress. **(A)**, H_2_O_2_ content; **(B)**, O_2_^·–^ production rate.

The contents of H_2_O_2_ and O_2_^•–^· in leaves of *T. chinensis* under saline-alkali stress were significantly higher than those in control (*p <* 0.05). The highest value was reached at the highest concentration (MSA4), which was 1.38-fold higher than that in control (**Figure 3A**). The O_2_^•–^· content reached a peak under MSA3 treatment (**Figure 3B**), which was 2.14-fold higher than that in control (*p <* 0.05), and then decreased significantly under MSA4 treatment (*p <* 0.05), with a decreasing rate of 34.03%.

### 3.4 Antioxidant enzyme activities

**Figure 4:**
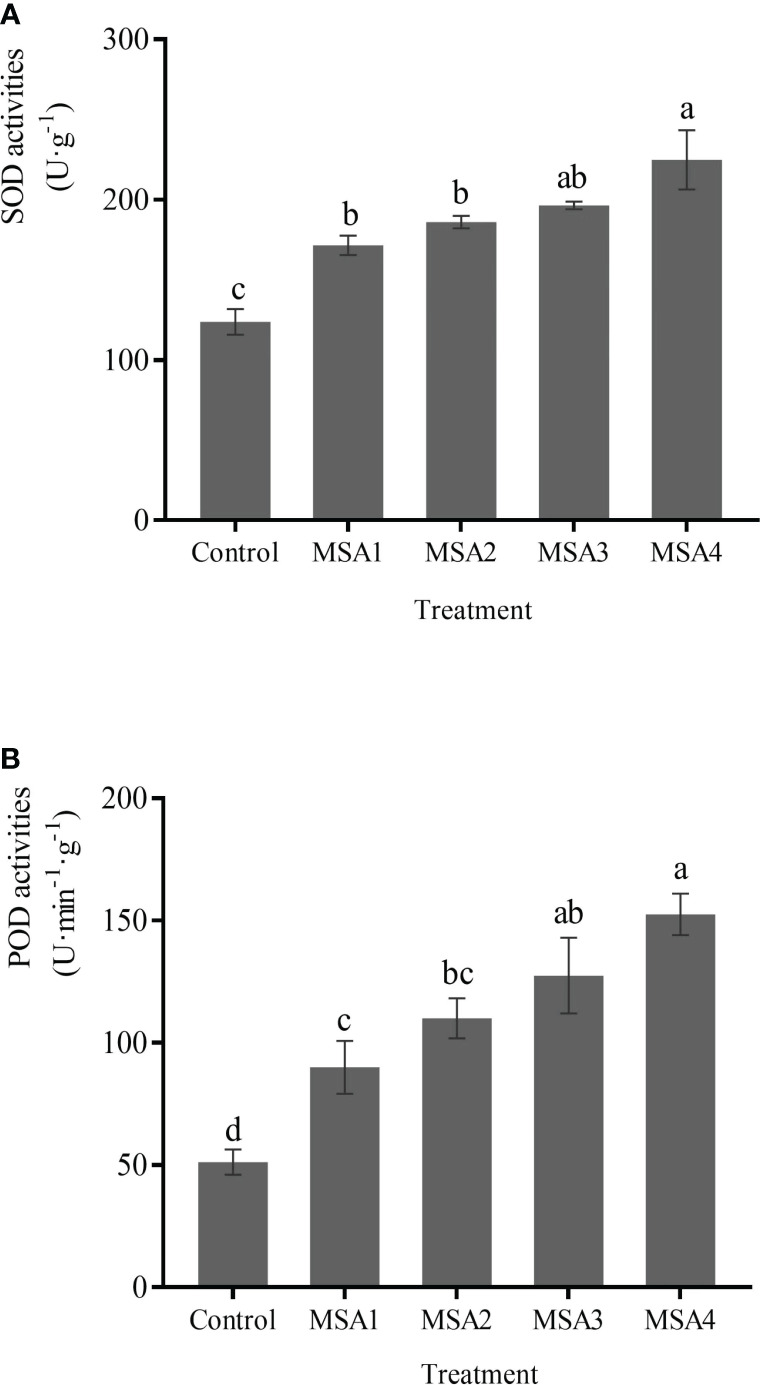
Changes in the activities of antioxidant enzymes in *Trollius chinensis* leaves upon saline-alkaline stress. **(A)**, SOD activities; **(B)**, POD activities.

The differences between low saline-alkali treatment group (MSA1 and MSA2) and high saline-alkali treatment (MSA3 and MSA4) group was not significant (*p > 0.05*). Compared with control, SOD activities increased by 38.68%, 50.30%, 58.77%, and 81.72% in MSA1, MSA2, MSA3, and MSA4, respectively (**Figure 4A**) while the activity of POD was 1.76, 2.15, 2.49 and 2.98-fold higher than that in control, respectively (**Figure 4B**).

### 3.5 AsA-GSH cycle

**Figure 5:**
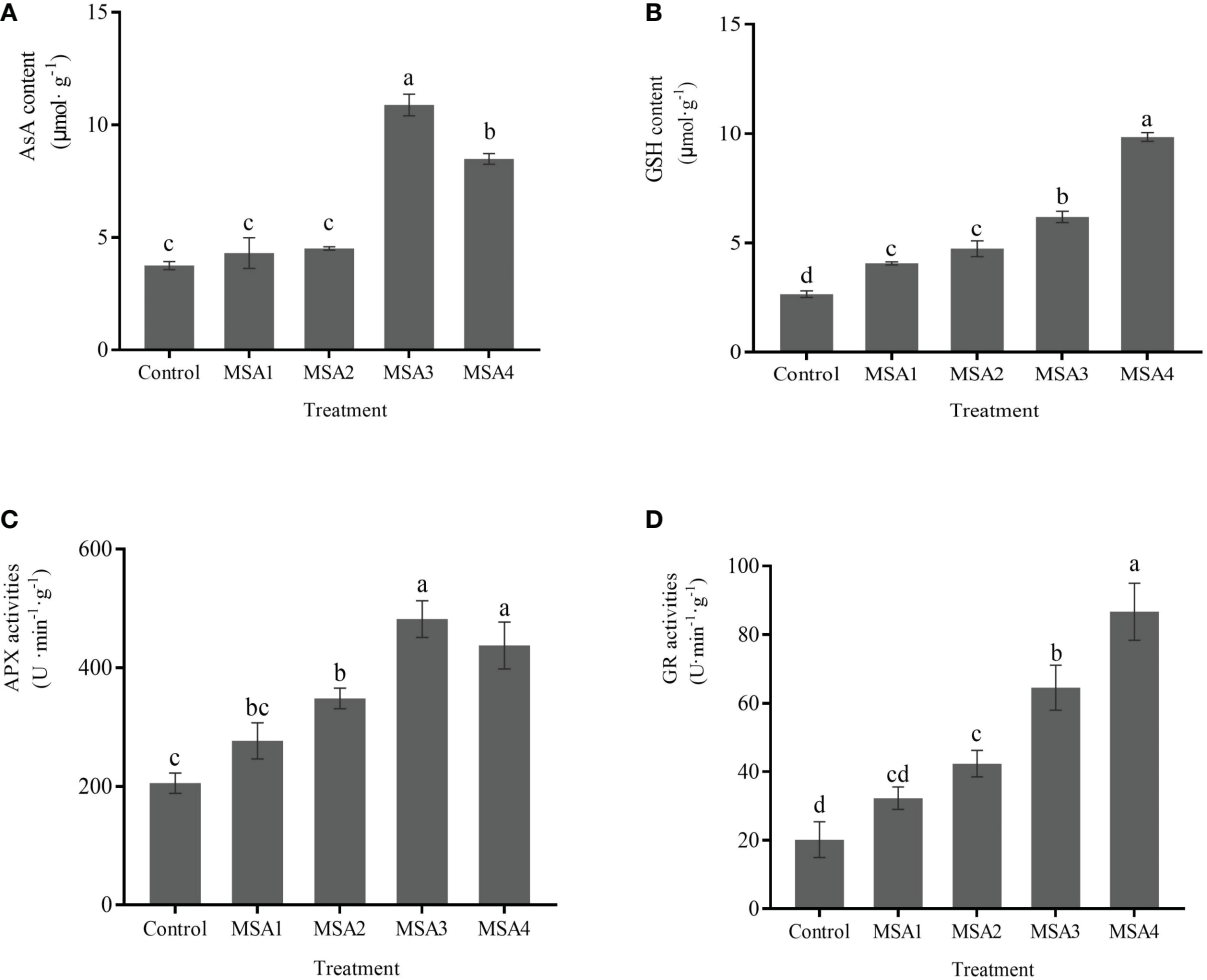
Response of AsA-GSH cycling system in *Trollius chinensis* leaves to saline-alkaline stress. **(A)**, AsA content; **(B)**, GSH content; **(C)**, APX activities; **(D)**, GR activities.

Compared with control, the contents of AsA and GSH and the activities of APX and GR increased to various degrees upon saline-alkali treatment, and the difference between high saline-alkali treatment (MSA3 and MSA4) and the low saline-alkali treatment (MSA1 and MSA2) groups was significant (*p <* 0.05). The changes of AsA and APX were consistent, and no significant difference was found between the low saline-alkali treatments (MSA1 and MSA2) (*p > 0.05*), and both reached the maximum value in the high saline-alkali treatment (MSA3) (**Figures 5A, C**), which were 2.90 and 2.35-fold higher than that in control, respectively (*p < 0.01*). While the changes of GSH and GR were consistent, and both reached the peak value at the highest concentration (MSA4), which were 3.71 and 4.30-fold higher than that in control, respectively (**Figures 5B, D**) (*p < 0.01*).

### 3.6 Correlation among different physiological characteristics

**Figure 6:**
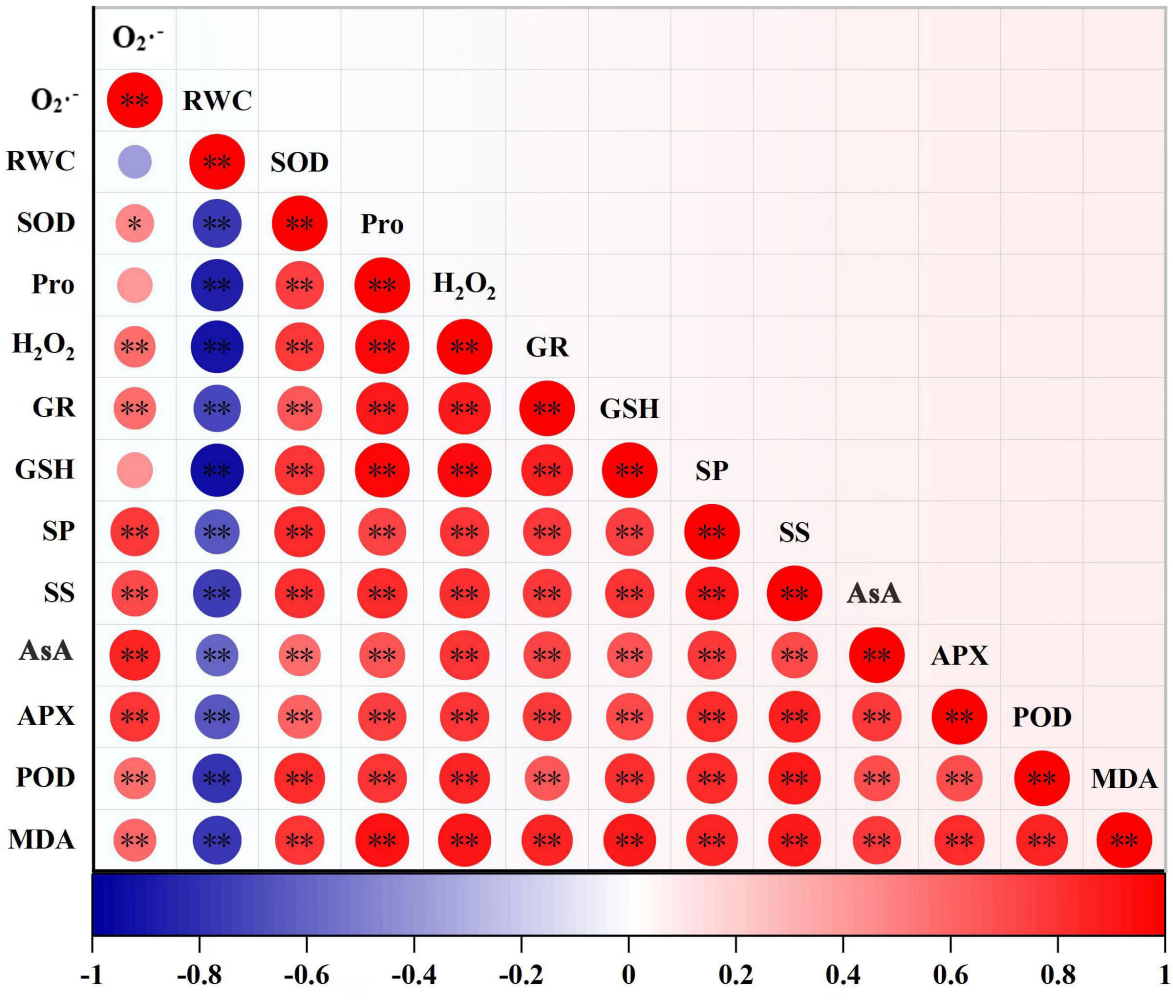
Pearson correlation between indicators. The deepness of color indicates the strength of the correlation, blue indicates negative correlation, and red indicates positive correlation, *p < 0.05, **p < 0.01. (RWC, leaf relative water content; MDA, malondialdehyde; Pro, proline; SS, soluble sugar; SP, soluble protein, O_2_^·–^, accumulation of superoxide anion; H_2_O_2_, hydrogen peroxide, SOD, superoxide dismutase; POD, peroxidase; AsA, ascorbic acid; GSH, glutathione; APX, ascorbate peroxidase; GR, glutathione reductase).

Among all indicators, the correlation analysis showed that only O^·–^ had no significant correlation with RWC, Pro and GSH (*p >* 0.05) while the remaining indicators were significantly correlated with each other (*p >* 0.05) (**Figure 6**). Among these significant correlation indicators, RWC was significantly negatively correlated with these indicators (*p <* 0.01), while the other indicators were significantly positively correlated (*p <* 0.01).

### 3.7 Principal component analysis of physiological characteristics

**Figure 7:**
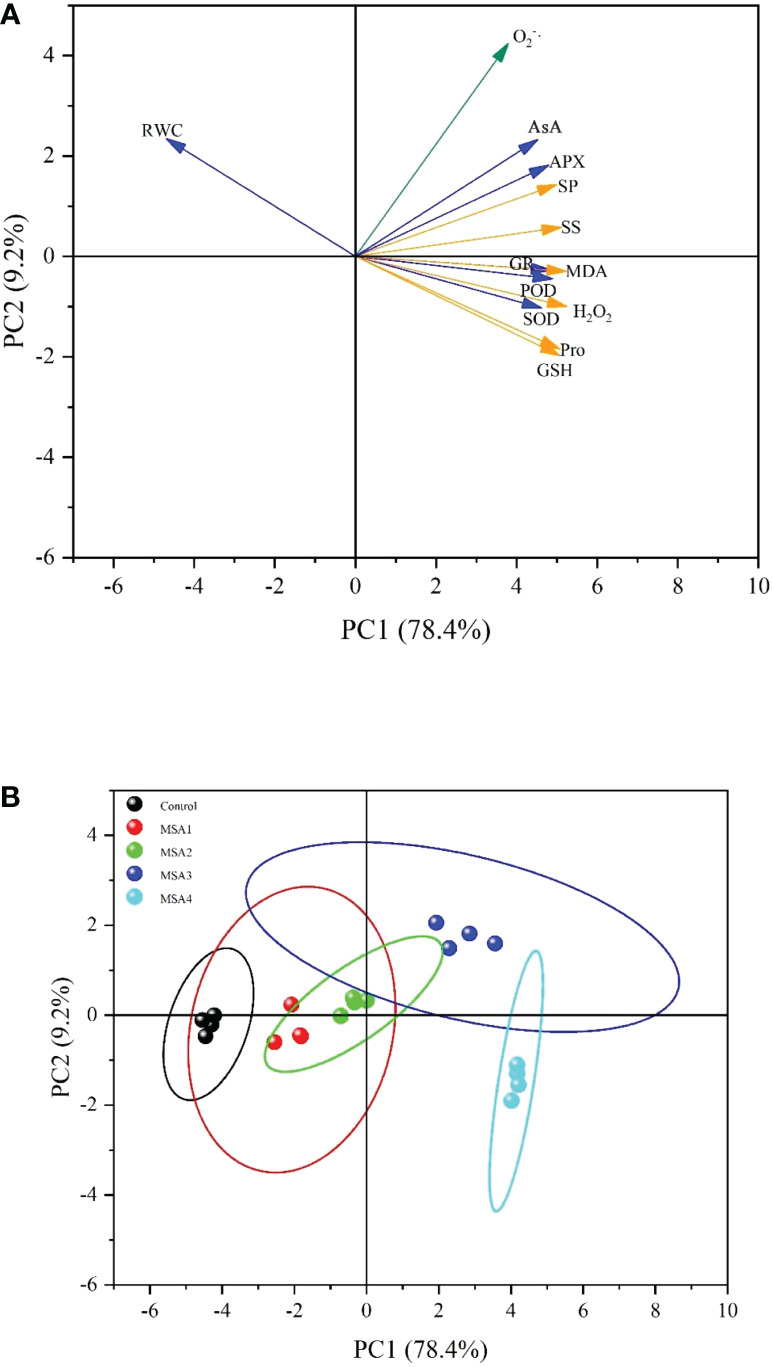
Principal component analysis of the measured parameters. **(A)**, Evaluation Indicator plot. (RWC, leaf relative water content; MDA, malondialdehyde; Pro, proline; SS, soluble sugar; SP, soluble protein, O_2_^-^·, accumulation of superoxide anion; H_2_O_2_, hydrogen peroxide, SOD, superoxide dismutase; POD, peroxidase; AsA, ascorbic acid; GSH, glutathione; APX, ascorbate peroxidase; GR, glutathione reductase); **(B)**, Score plot of each group. (MSA1: 40 mM; MSA2: 80 mM; MSA3: 120 mM; MSA4: 160 mM).

The PCA of the physiological characteristics proved that the first component (PC1, 78.4%) and second component (PC2, 9.2%) explained 87.6% of the total variance (**Figure 7**). In PC1, RWC was a negative marker, the rest were positive markers, and the load weights of H_2_O_2_, MDA, GSH, Pro, SS and SP were the highest, indicating that the PC1 reflects the ability of seedling osmotic regulation. There were many negative markers in PC2, and O_2_^·–^ loading weight was the highest, which proves that PC2 mainly reflects the relationship between ROS response and saline-alkali stress (**Figure 7A**). Combined with the extracted PC, H_2_O_2_, MDA, GSH, Pro, SS, SP and O_2_^·–^ were well representative and could be used as the evaluation index of osmotic antioxidant regulation ability of *T. chinensis* under saline-alkali stress. The scores were calculated for each treatment (**Figure 7B**), the difference between the samples under low saline-alkali treatment and control was not obvious, but the difference between the samples under high saline-alkali group and control was significant. The most significant effect of saline-alkali stress on osmotic regulation and antioxidant capacity of *T. chinensis* is MSA3 (120 mM), followed by MSA4 (160 mM), MSA2 (80 mM), and MSA1 (40 mM).

### 3.8 General information of transcriptome sequencing and assembly

A total of 36.05 Gb of clean sequences were obtained, with a percentage of Q30 bases in each sample above 93% and a GC content above 44% (**Table S2**). This result indicated the quality of RNA-Seq datasets was high. Assembly resulted in a total of 66,954 unigenes, with an N50 length of 1,525 bp and a mean length of 563 bp. The length distribution of the assembled unigenes is shown (**Figure S1**).

### 3.9 Identification of DEGs

In total, 474 (364 up- and 110 down-regulated) DEGs was identified in MSA1 group *vs*. control. Based on the expression levels of the DEGs (using the log_10_(FPKM+1) value), hierarchical clustering analysis was performed (**Figure S2**). Gene expression patterns in clusters with similar colors are similar, indicating that these genes may have similar functions or participate in regulating the same metabolic pathway.

### 3.10 GO enrichment of the DEGs

**Figure 8:**
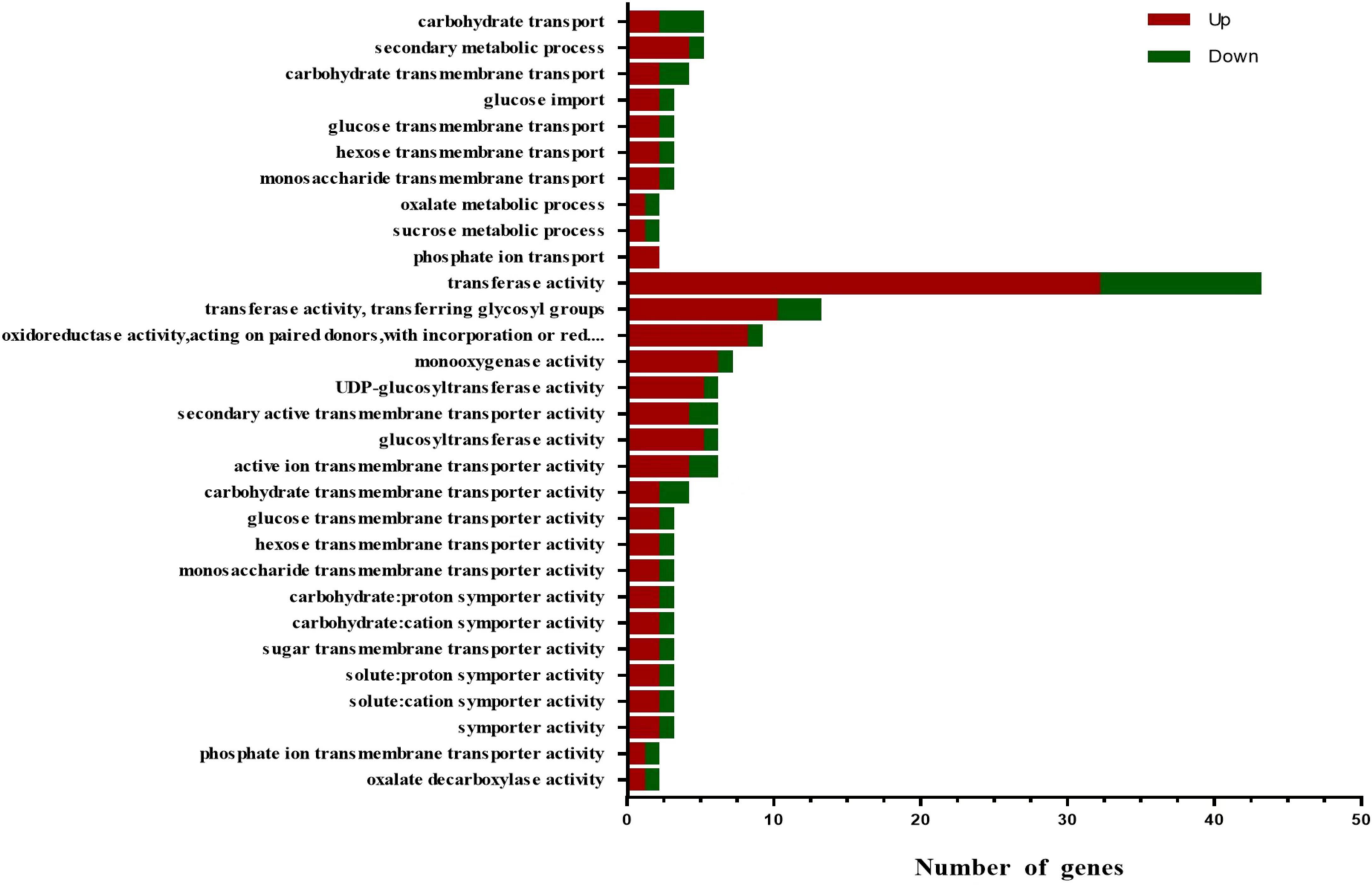
Gene Ontology (GO) analysis of differentially expressed genes. Red indicates genes with increased expression, and green indicates genes with decreased expression. BP, biological processes; MF, molecular function.

GO enrichment analysis was performed on the DEGs, and the functions were enriched and classified according to the three categories of biological processes, cellular components, and molecular functions. The top 30 functions were screened (*p <* 0.05) (**Figure 8**). It showed that only two categories of biological processes (BP) and molecular functions (MF) are enriched. A total of 32 DEGs were enriched in the biological process, including 20 up-regulated and 12 down-regulated DEGs. Many DEGs were enriched in ‘carbohydrate transport’, ‘secondary metabolic process’ and ‘carbohydrate transmembrane transport’. In total, 131 DEGs were enriched in molecular function, of which 96 were up-regulated and 35 were down-regulated, especially in the process of ‘transferase activity’, followed by ‘transferase activity, transferring glycosyl groups’, and ‘oxidoreductase activity’.

### 3.11 KEGG pathways enrichment of the DEGs

The DEGs were divided into five branches according to the KEGG metabolic pathways, including cellular processes, environmental information processing, genetic information processing, metabolism and organismal system. Many DEGs were enriched in metabolic pathways (69 DEGs), followed by genetic information processing (17 DEGs). The enriched metabolic pathways were closely related to saline-alkali stress (**Table S3**). For example, the ‘zeatin biosynthesis’ pathway was the most significant enriched, including two down-regulated genes and one up-regulated gene; followed by ‘ABC transporters’, in which three DEGs were up-regulated; then the ‘spliceosome’, including three up-regulated genes and two down-regulated genes. Other pathways were mainly involved in metabolism, such as ‘isoflavonoid biosynthesis’, ‘tyrosine metabolism’, and ‘terpenoid backbone biosynthesis’.

### 3.12 DEGs related to osmoregulation

Saline-alkali stress affected the expression of genes which took part in osmotic synthesis, sucrose synthesis, amino acid metabolism, and other processes in *T. chinensis* seedlings. These gene encode betaine aldehyde dehydrogenase, IMP biosynthetic process, sucrose synthase, tyrosine metabolic process, sucrose phosphatase and annexin. Among them, four genes were up-regulated and two were down-regulated (**Table 2**), indicating that under low-saline-alkali stress, plants could probably respond to stress by synthesizing small molecules of soluble sugars and proteins through metabolic regulation, thereby improving its resistance.

**Table 2:** Differentially expressed genes related to osmoregulation.

Gene ID	Log_2_FC	Gene annotation
*TRINITY_DN26035_c2_g3*	1.45	Betaine aldehyde dehydrogenase
*TRINITY_DN25850_c0_g1*	-1.79	IMP biosynthetic process
*TRINITY_DN23089_c1_g2*	4.36	Sucrose synthase
*TRINITY_DN26557_c0_g1*	2.67	Tyrosine metabolism
*TRINITY_DN25801_c1_g5*	-3.24	Sucrose-phosphatase
*TRINITY_DN23416_c7_g1*	3.89	Annexin

### 3.13 DEGs related to antioxidant system

In terms of antioxidants, four and three DEGs related to chloroperoxidase (CPO), germin-like proteins (GLP), thioredoxin (Trx), glutaredoxin, glutathione metabolism, and ascorbate family oxidoreductases (DLO) were up- and down-regulated, respectively (**Table 3**). It probably means that *T. chinensis* seedlings could positively initiate the antioxidant regulation *in vivo* and maintain the balance of antioxidant system under low saline-alkali stress.

**Table 3:** Differentially expressed genes related to antioxidant enzymes.

Gene ID	Log_2_FC	Gene annotation
*TRINITY_DN17203_c0_g1*	5.30	Chloroperoxidase
*TRINITY_DN25930_c6_g1*	-2.74	Germin-like protein subfamily
*TRINITY_DN25275_c1_g3*	1.74	Germin-like protein subfamily
*TRINITY_DN22556_c0_g1*	2.38	Thioredoxin
*TRINITY_DN20143_c0_g1*	2.67	Glutaredoxin
*TRINITY_DN21144_c0_g2*	-2.15	Glutathione metabolism
*TRINITY_DN22122_c0_g1*	-1.29	2OG-oxygenase-like protein DLO

### 3.14 Transcription factors

**Figure 9:**
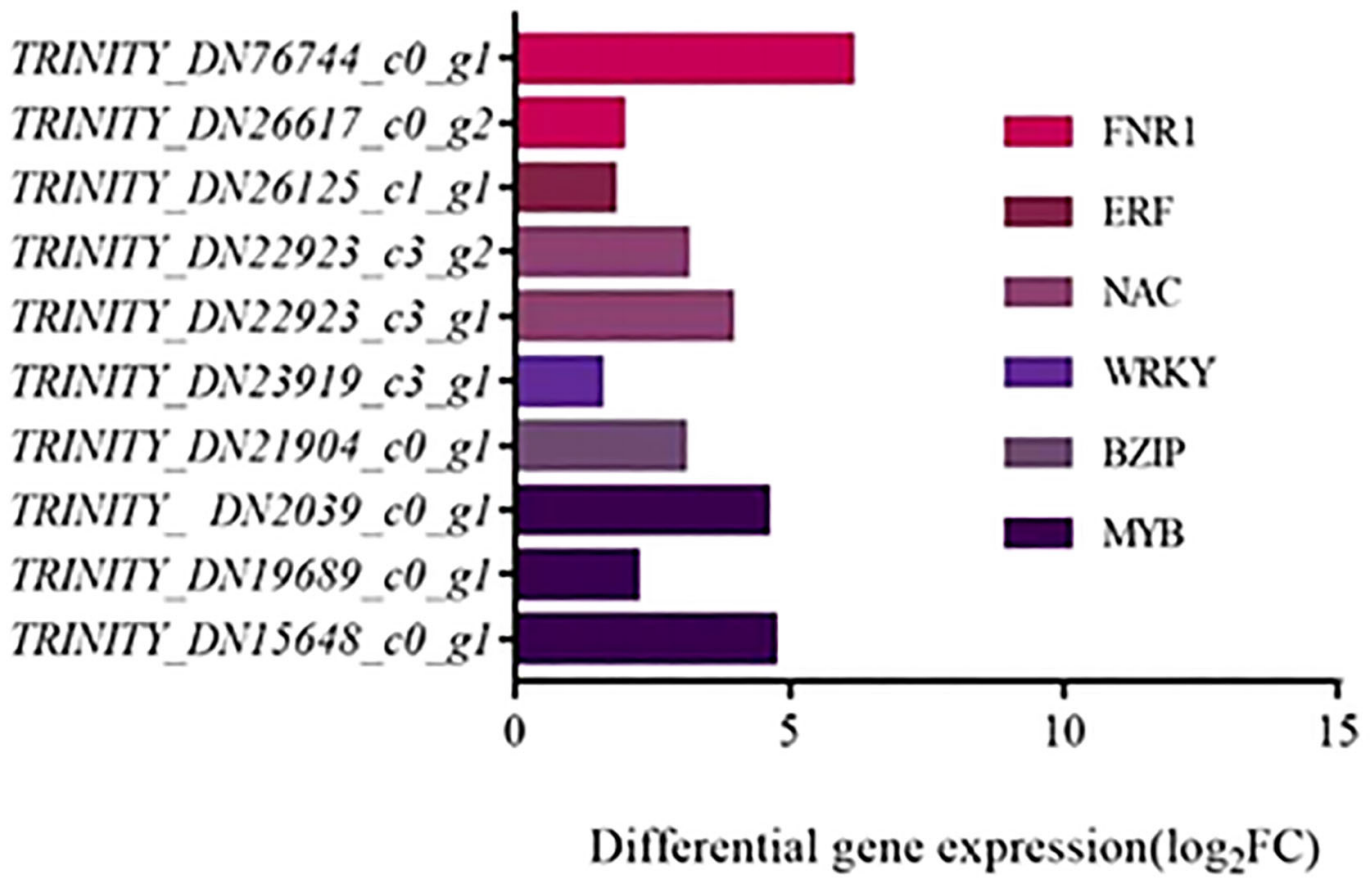
Transcription factors with differential expressions and their corresponding families.

According to the transcriptome results, some differentially expressed transcription factors (TFs) were screened for saline-alkali tolerance, mainly including 10 DEGs belonging to MYB, BZIP, WRKY, NAC, ERF and FNR1 families (**Figure 9**). All these DEGs were all up-regulated, which proves that these transcription factors have positive regulatory effects on the response of *T. chinensis* to saline-alkali stress. Among them, three DEGs belonging to MYB family, two DEGs to either NAC or FNR1 families, and each of other transcription factor families had one related DEG. The DEG (*TRINITY_DN76744_c0_g1*) belongs to FNR1 family had the highest expression level, while the DEG (*TRINITY_DN23919_c3_g1*) of the WRKY family had the lowest expression.

### 3.15 The analysis of quantitative real-time PCR

**Figure 10:**
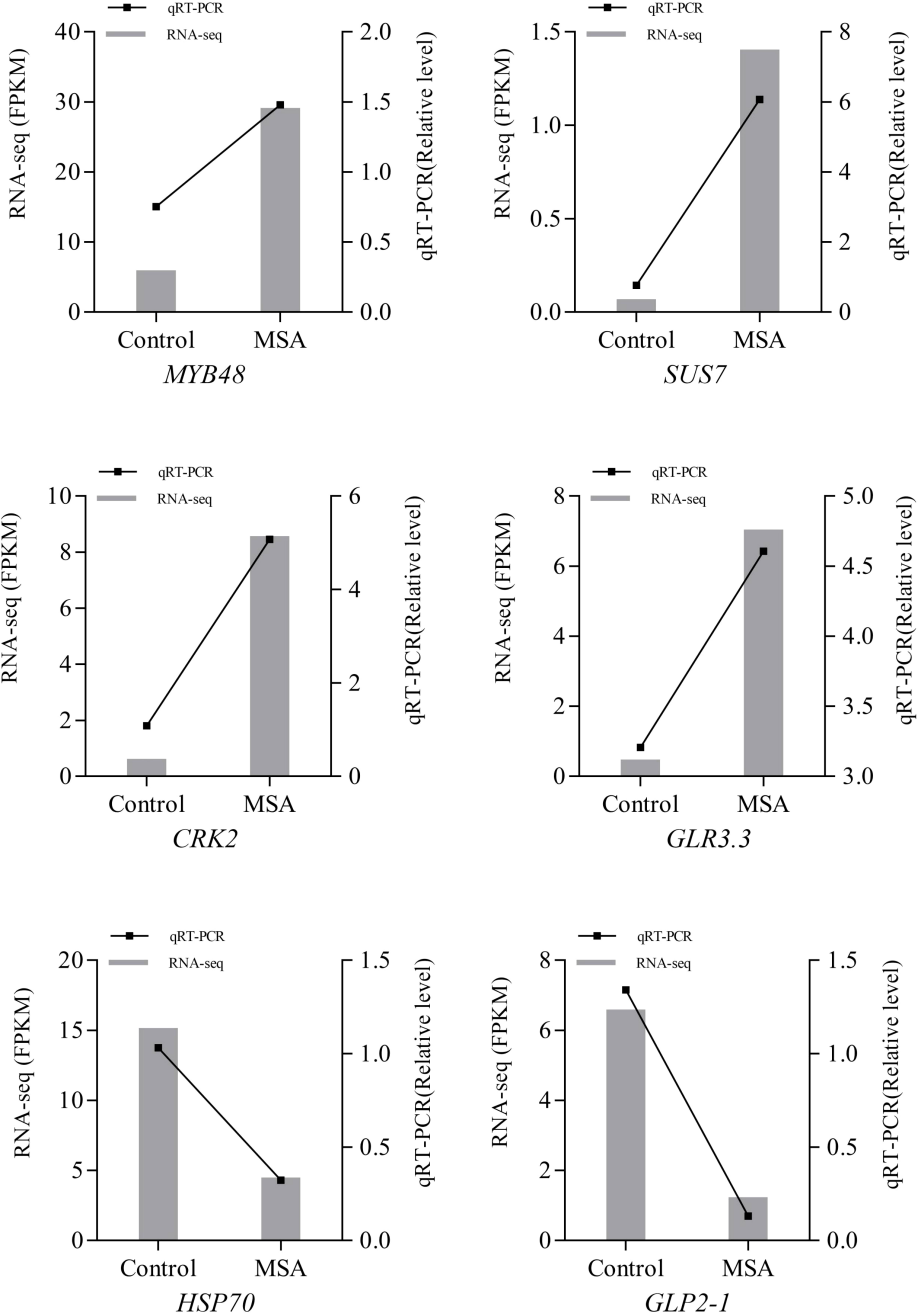
Quantitative Real-Time PCR (qRT-PCR) validation of differential expression genes. Control, 0 mM; MSA, 40 mM. *MYB48*, transcription factor MYB48; *SUS7*, Sucrose synthase 7-like; *CRK2*, Cysteine-rich receptor-like protein kinase 2; *GLR3.3*, Glutamate receptor 3.3; *HSP70*, Heat shock 70 kDa protein; *GLP2-1*, germin-like protein 2-1.

To verify the accuracy of the RNA-Seq results, genes related to saline-alkali stress were selected for qRT-PCR, including DEGs encoding stress functional protein (HSP70), antioxidant protein (GLP2-1), and plant receptor protein kinase (CRK2), and sucrose synthase (SUS), transcription factor (MYB48), and ion channel receptor (glutamate receptor‐like, GLR3.3). It is showed that the qRT-PCR results were consistent with the RNA-Seq data (**Figure 10**).

## 4 Discussion

By altering water permeability, cellular turgidity and water potential, excessive salt induces osmotic stress (Raziq et al., 2022), which in turn leads to inhibition of physiological activity. In the present study, the RWC of *T. chinensis* leaves under low saline-alkali conditions was not significantly different compared to control, which probably indicated that seedlings were tolerant to low saline-alkali level (**Figure 1A**). However, under high-saline-alkali conditions water loss of the leaves was severe. The decline was mostly attributable to hyperosmotic stress brought on by an increase in saline-alkali concentration. This reduction in RWC is the key sign of salt stress, which restricts water flow to the sites of new cell elongation, and is consistent with tomato research (Raziq et al., 2022) and *Aloe vera* (Nakhaie et al., 2020). Moreover, the oxidative stress induced by saline-alkali stress led to lipid peroxidation and MDA buildup. Under high saline-alkali stress, MDA content in *T. chinensis* leaves was higher than that under low saline-alkali stress, suggesting the greater oxidative damage to the plasma membrane.

Osmoregulation is an important mechanism for enhancing stress resistance in plants (Hannachi and Van Labeke, 2018). Plants can maintain osmotic balance by accumulating organic substances called osmolytes such as osmotic agent Pro, SS, betaine, etc. to improve salinity tolerance (Shi and Sheng, 2005; Sperdouli and Moustakas, 2012). Our study demonstrated that saline-alkali stress markedly increased the contents of osmoregulatory substances in *T. chinensis* leaves, even in low saline-alkali environment, which should be a self-protection of seedlings As an effective osmoprotectant, SS can enhance the osmotic potential of plant cells to maintain ion homoeostasis (Qiu et al., 2017). In the present study, saline-alkali stress significantly increased the accumulation of SS content in *T. chinensis* leaves (**Figure 2B**), which is consistent with the change in Pro content (**Figure 2A**). Protein synthesis is one potential primary target of saline toxicity. Earlier studies have indicated that SP for responding tend to differ according to plant species, as well as variety, development, and saline exposure length and degree (Wang et al., 2020). For example, the SP content of *Sorghum bicolor* leaves increased first and then decreased with the increase of pH value under saline-alkali stress (Sun et al., 2019), but the SP content of maize (Hamdia et al., 2004) increased with higher degree of salt stress. Similar to these findings, we found that the content of SP in *T. chinensis* leaves first increased and then decreased slightly with the increase of salinity (**Figure 2C**). It may be due to the SP in seedlings had a certain threshold in response to saline-alkali stress, and high salt concentration had a certain inhibition on the accumulation of SP. At the transcriptome level, some DEGs were enriched in KEGG pathways associated with the osmotic regulation such as sucrose synthesis and amino acid metabolism, and most of the genes involved in these metabolic pathways were up-regulated under saline-alkali stress. The expression of betaine aldehyde dehydrogenase gene (*BADH*) was up-regulated to catalyze the production of betaine from choline in the seedlings of *T. chinensis*, thereby protecting the activity of intracellular proteins and metabolic enzymes and stabilizing the cell membrane (Tian et al., 2017). In the process of sucrose metabolism, the up-regulated sucrose synthase gene (*SUS*) could encode a synthase that promoted sucrose synthesis and decomposition (Maloney et al., 2015).

ROS homeostasis in plants is regulated by complex enzymatic and non-enzymatic antioxidant defense system (Sugimoto et al., 2014). When plants are subjected to saline-alkali stress, a large amount of ROS will accumulate, disrupting the original balance. As a stress signal, it can trigger protecting enzymes such as SOD, POD, and APX, and limit the oxidative damage caused by excessive ROS (Ahanger et al., 2018). In the present study, after *T. chinensis* seedlings were subjected to saline-alkali stress, H_2_O_2_ continued to increase, and O_2_^·–^ content first increased and then decreased (**Figure 3**). We speculate that O_2_^·–^ might be produced in large quantities in the early stage, and then converted into H_2_O_2_ for further accumulation. These results are similar to the study of alfalfa (An et al., 2016). In the same time, SOD as the first line of defense, catalyzes the conversion of O_2_^·–^ into and O_2_, and POD catalyzes the conversion of H_2_O_2_ into O_2_ and H_2_O (Meloni et al., 2003). We found that SOD and POD were activated by saline-alkali stress, and their activities were continuously enhanced to cope with oxidative damage of ROS (**Figure 4**). Moreover, the AsA-GSH cycle that aids enzyme against oxidation in scavenging excess ROS, is a crucial antioxidant-mediated protection mechanism in plants. AsA and GSH are both potent antioxidants aiding the plasma membrane against oxidation caused by stress by acting as buffers in redox processes (Foyer and Noctor, 2011). AsA acts as an electron donor for APX to scavenge H_2_O_2_ (Drązkiewicz et al, 2003), while GR acts as a rate-limiting enzyme in the ROS scavenging system, which can increase and maintain enhanced GSH content in plants (Moradi and Ismail, 2007). In the present study, the contents or activities of AsA, APX, GSH, and GR were maintained or increased in various degrees after *T. chinensis* seedlings were subjected to saline-alkali stress (**Figure 5**), among which AsA and APX showed a downward trend under high concentrations, similar to the findings of *Lolium perenne* (Ma et al., 2016). In our study, the increase of GR activity after saline-alkali stress elevated the GSH content (**Figures 5B, D**), which was then reduced to AsA through the AsA-GSH cycle. It indicated that the content of AsA and APX activity were still significantly higher than the control though they were slightly decreased under high saline-alkali concentration (**Figures 5A, C**). After GSH is oxidized, it can be recycled by GR again, which proves that *T. chinensis* seedlings tried to maintain redox regulation under severe saline-alkali stress. While the contents or activities of AsA, GR, and APX did not change significantly under MSA1, suggesting that the oxidative damage to the seedlings was slight and plants could maintain normal physiological activities. This result is consistent with the transcriptomic data. *T. chinensis* seedlings promoted the scavenging of ROS by increasing the expression of genes encoding peroxidase and thioredoxin. Among them, peroxidase genes can regulate the activity of antioxidant enzymes, and thioredoxin can participate in the salt tolerance of plants through redox regulation (Boubakri et al., 2019). It has been reported that germin-like proteins (GLPs) take part in plant response to abiotic stresses involving hydrogen peroxide (H_2_O_2_) production (Gangadhar et al., 2021). In the present study, the down-regulated expression of gene encoding germin-like protein 2-1 probably inhibited the production of H_2_O_2_ to alleviate the oxidative damage caused by saline-alkali stress.

Transcription factors regulate gene expression concerning metabolism among plant cells, and also play crucial roles in stress response (An et al., 2016). Currently, many families of salt-tolerant transcription factors MYB, WRKY, bZIP, etc. have been confirmed (Luo et al., 2016). In the present study, six transcription factor families were mainly screened, among which the DEGs belonging to MYB, NAC and FAR1 families had high expression under stress. In our study, the expression of three MYB transcription factors were significantly up-regulated under stress, which is consistent with previous studies in which MYB in Arabidopsis and soybean were strongly up-regulated in response to salinity (Liao et al., 2008; Xu et al., 2015). The NAC family is one of the largest transcription factor families in plants, and it reacts to a variety of stressors (Nakashima et al., 2007). The expression levels of the two NAC transcription factors in our study were similar and both were up-regulated, which was consistent with the results of the alfalfa study (An et al., 2016). In addition, two FAR1 family transcription factors were differentially expressed, and one of them had the highest expression level among all transcription factors in the present study, which is similar to a previous study that transcription factor *FAR1* of *Aloe vera* was significantly up-regulated under saline-alkali stress (Nayak et al., 2020). The high expression level of *FAR1* members may indicate that *FAR1* plays an important role in the tolerance to saline-alkali stress of *T. chinensis.* Many studies have found that overexpression of transcription factors can regulate plant stress resistance. For example, overexpressing of *OsMYB48* in rice inhibited the water loss rate and MDA content, and increased Pro content under salt stress conditions (Xiong et al., 2014). The overexpression of *OsNAC10*, *OsbZIP23* and *OsbZIP46* can also significantly improve the drought and high salt stress in rice (Jeong et al., 2010; Tang et al., 2012). The overexpression of *GhWRKY39* in cotton increases the tolerance of transgenic plants to salt oxidative stress and improves the activities of antioxidant enzymes (SOD and POD) (Shi et al., 2014). Therefore, we suspect that the transcription factors excavated in the current study such as NAC and FAR1 may play key roles in regulating the tolerance of *T. chinensis* to saline-alkali stress, which need to be further studied.

Plant GLR members has been considered as Ca^2+^ channels and play key roles in many physiological processes (Kong et al., 2016). *GLR3.3*, screened as a gene for qRT-PCR in this study, is one of the common genes in the glutamate receptors. In Arabidopsis, *GLR3.3* is required for GSH‐mediated innate immunity response, suggesting a crosstalk of GSH and GLRs in defense signaling (Li et al., 2013). Scientists also studied the functions of *GLR3.3/GLR3.5* in tomato and found that *GLR3.3/GLR3.5* played important roles in the H_2_O_2_‐GSH/GSSG‐dependent signaling pathway, which is important for cold acclimation‐induced chilling tolerance (Li et al., 2019). In the present study, the *GLR3.3* and *GLR3.5* genes were also up-regulated by saline-alkali stress, and we speculate that GLR3.3/GLR3.5 probably promoted Ca^2+^ influx and regulated H_2_O_2_ production, which affected the ROS signaling and the subsequent expression changes of target genes related with osmotic regulation and antioxidant response

## 5 Conclusion

**Figure 11:**
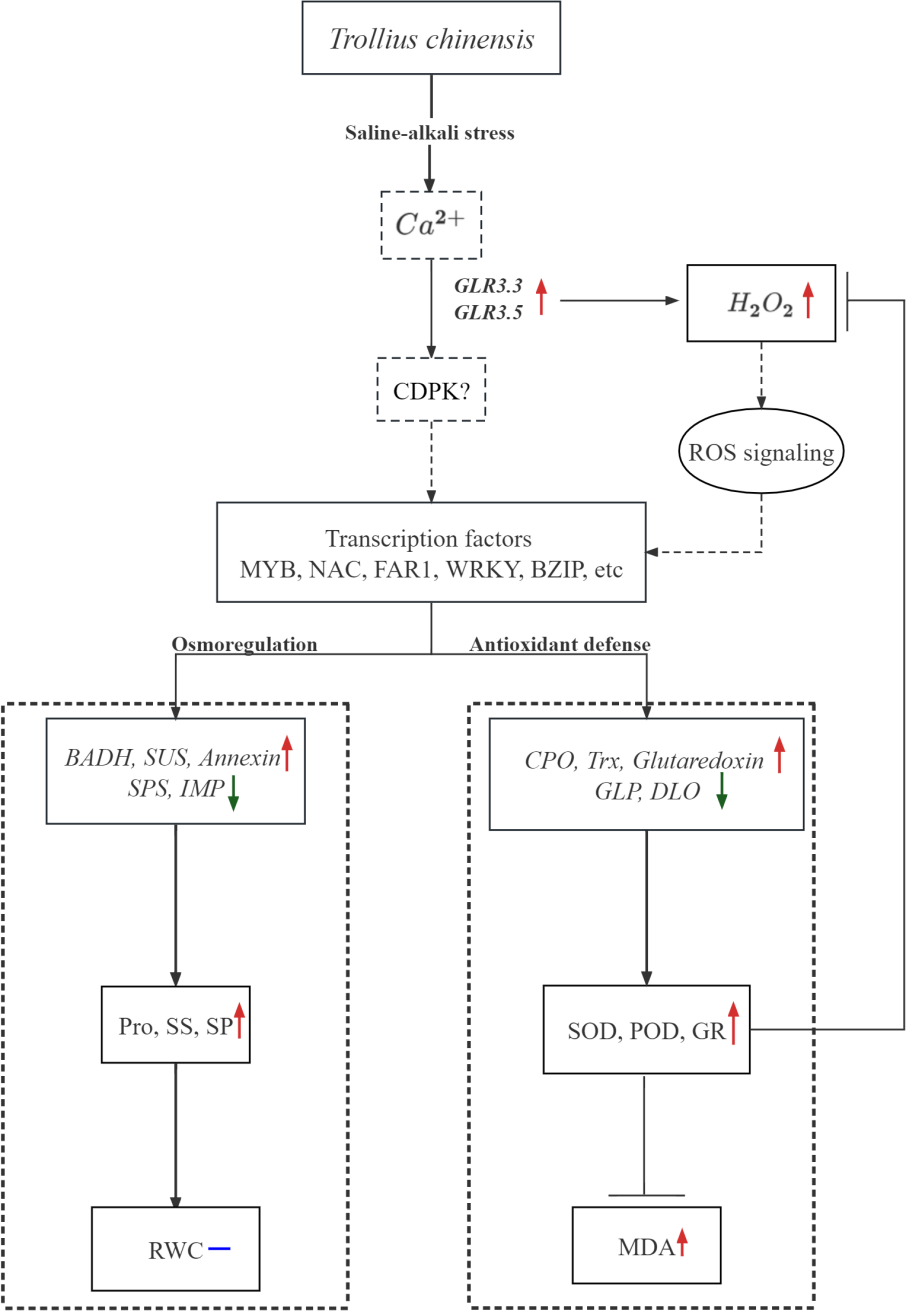
The work model of *Trollius chinensis* in response to low saline-alkali stress (MSA1, 40mM). Red arrows indicate genes with increased expression or substances with increased content or activity, green arrows indicate genes with decreased expression or substances with decreased content or activity, and blue dash line represents that the content of certain substance was not changed.

This study firstly reported a comprehensive physiological and transcriptomic analysis about the responses of *T. chinensis* to saline-alkali stress. In *T. chinensis* seedlings under saline-alkali stress, GLR- dependent influx of Ca^2+^ was enhanced, the H_2_O_2_ was accumulated and the ROS signaling pathway activated, then many transcription factors belonging to MYB, NAC family were regulated. After that, the expressions of some genes related with osmotic regulation and antioxidant processes were significantly changed. Therefore, osmotic regulation was activated to produce substances to maintain the membrane stability, and antioxidant systems were also activated to inhibit the ROS accumulation and the MDA content (**Figure 11**). Additionally, in our study, we observed that some of the indicators, such as RWC, could be used as markers for salinity and alkalinity tolerance of this species. Based on the relative changes of above characteristics, it is concluded that *T. chinensis* was slightly affected by low saline-alkali, but much deeply affected by high saline-alkali stress (120 mM). All these results provide clues for future exploration of the molecular mechanisms of *T. chinensis* tolerance in response to salinity and alkalinity.

## Data availability statement

The data presented in the study are deposited in the NCBI SRA repository, accession number PRJNA893832.

## Author contributions

LZ and RH designed the study. RH drafted the manuscript and analyzed the RNA-seq data. RH and LY completed the main experiments in this work. SC participated in data analysis. LZ and TW provided guidance on data analysis and contributed to the revision of the manuscript. All authors contributed to the article and approved the submitted version.
