# Supplementary figures and images for: Genes related to osmoregulation and antioxidation play important roles in the response of Trollius chinensis seedlings to saline-alkali stress
Source: Front Plant Sci. 2023 Jan 26;14:1080504. doi: 10.3389/fpls.2023.1080504 (PMC9911134; doi:10.3389/fpls.2023.1080504)

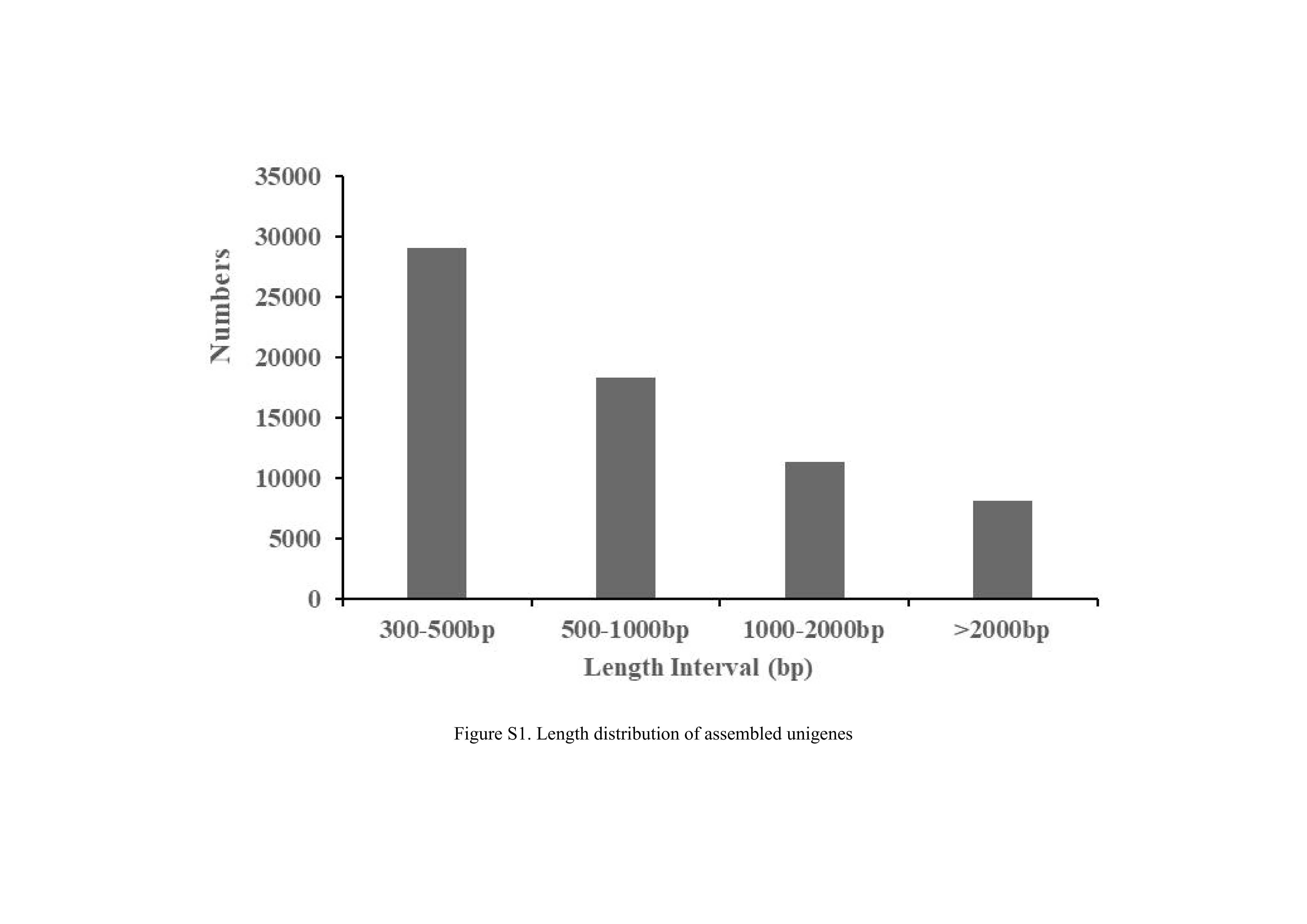

Supplement: Supplementary file 1 [file Image_1.jpg]

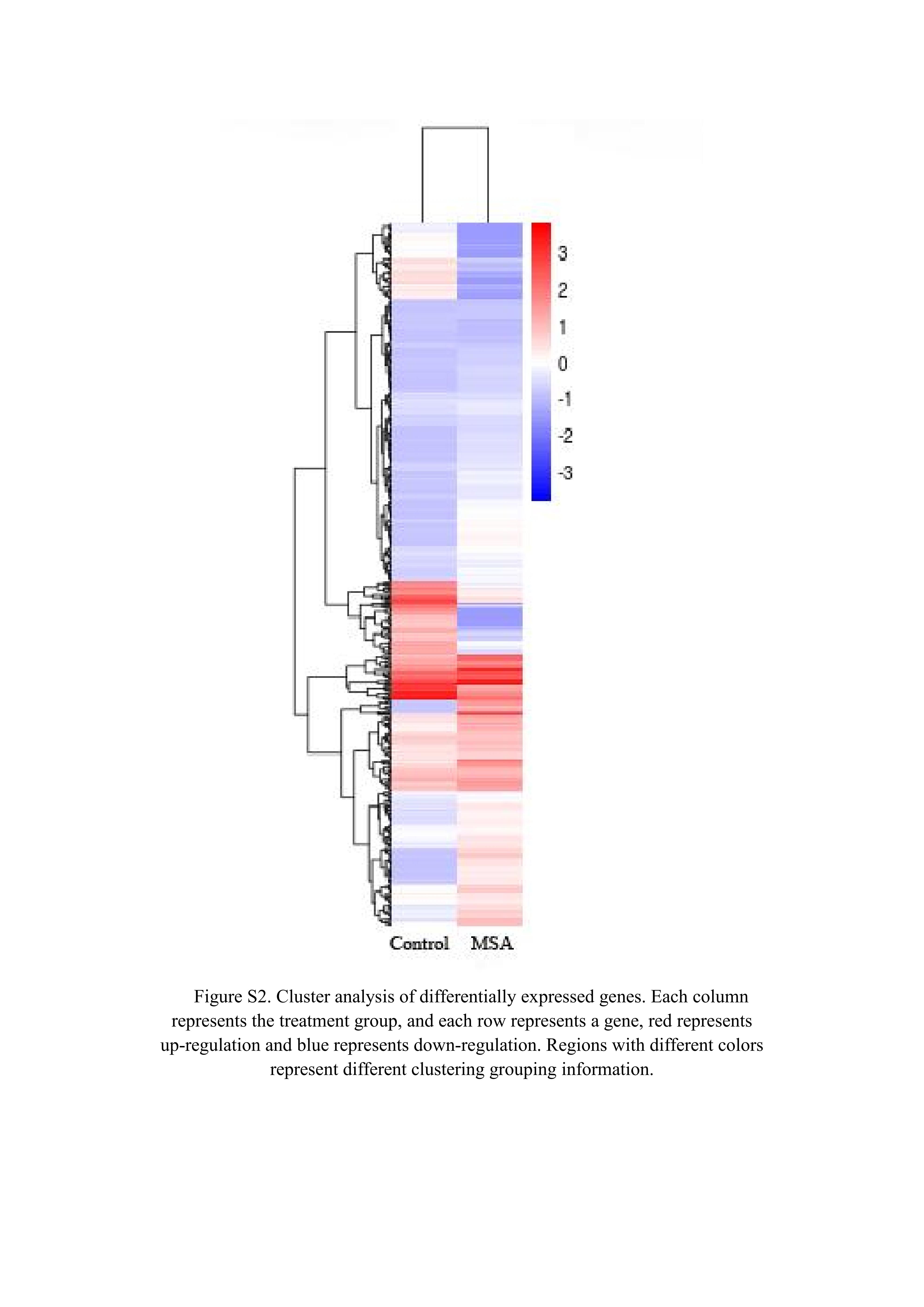

Supplement: Supplementary file 2 [file Image_2.jpg]
