# Supplementary material for: Genes related to osmoregulation and antioxidation play important roles in the response of Trollius chinensis seedlings to saline-alkali stress
Source: Front Plant Sci. 2023 Jan 26;14:1080504. doi: 10.3389/fpls.2023.1080504 (PMC9911134; doi:10.3389/fpls.2023.1080504)
Supplement: Supplementary file 3 [file Table_1.docx]

Supplementary Table S1. qRT-PCR primer sequences

| Gene ID | Gene annotation | Primer F | Primer R |
| --- | --- | --- | --- |
| *MYB48* | Transcription factor MYB48 | GAAAAGGAGTCCACAGCACC | AGCTATGTAATCCCATCGTC |
| *SUS7* | Sucrose synthase 7-like isoform X6 | TCACATCCTCCGTGTTCCAT | GACGCCACCAAGTTACCATC |
| *CRK2* | Cysteine-rich receptor-like protein kinase 2 | CTACTCGGATAGTCTTGTCA | TACCCTCTGGATCTCCTGCT |
| *GLR3.3* | Glutamate receptor 3.3 | GCTTCTTCACAGGACGACAA | GGGTGGTTTAGAGCGAGTTG |
| *HSP70* | Heat shock 70 kDa protein | AGCGGTGAAAGCAACGTAAG | TGTAGGTGAAGGGGAAGGTA |
| *GLP2-1* | Putative germin-like protein 2-1 | CCTTGTTGCTTTCACCCTAC | TCATCTCCAGTAACAGTCGC |
| *Actin* | Reference gene | TCTTGACTACGAGCAGGAGC | GGTAGAACCACCACTGAGAA |
