# Supplementary material for: Genes related to osmoregulation and antioxidation play important roles in the response of Trollius chinensis seedlings to saline-alkali stress
Source: Front Plant Sci. 2023 Jan 26;14:1080504. doi: 10.3389/fpls.2023.1080504 (PMC9911134; doi:10.3389/fpls.2023.1080504)
Supplement: Supplementary file 4 [file Table_2.docx]

Supplementary Table S2. Statistics of sequencing data quality

| Samples | Total clean Reads | Error Rate | GC content（%） | Q30 value（%） |
| --- | --- | --- | --- | --- |
| Control | 39,392,930 | 0.03% | 45.05% | 93.21% |
| MSA | 40,747,749 | 0.03% | 44.74% | 93.27% |
