# Supplementary material for: Genes related to osmoregulation and antioxidation play important roles in the response of Trollius chinensis seedlings to saline-alkali stress
Source: Front Plant Sci. 2023 Jan 26;14:1080504. doi: 10.3389/fpls.2023.1080504 (PMC9911134; doi:10.3389/fpls.2023.1080504)
Supplement: Supplementary file 5 [file Table_3.docx]

Supplementary Table S3. Top 10 pathways with the most significant DEGs enrichment

| Pathway name | KO ID | DEGs in the pathway with annotation |
| --- | --- | --- |
| Zeatin biosynthesis | ko00908 | 0.31% |
| ABC transporters | ko02010 | 0.39% |
| Spliceosome | ko03040 | 2.02% |
| Isoflavonoid biosynthesis | ko00943 | 0.09% |
| Tyrosine metabolism | ko00350 | 0.66% |
| Terpenoid backbone biosynthesis | ko00900 | 0.68% |
| Brassinosteroid biosynthesis | ko00905 | 0.16% |
| MAPK signaling pathway - plant | ko04016 | 1.73% |
| Glycosaminoglycan degradation | ko00531 | 0.18% |
| Endocytosis | ko04144 | 1.78% |
